# Implementing the Bruker MALDI Biotyper in the Public Health Laboratory for *C. botulinum* Neurotoxin Detection

**DOI:** 10.3390/toxins9030094

**Published:** 2017-03-09

**Authors:** Michael J. Perry, Dominick A. Centurioni, Stephen W. Davis, George E. Hannett, Kimberlee A. Musser, Christina T. Egan

**Affiliations:** 1 Biodefense Laboratory, Wadsworth Center, New York State Department of Health, 120 New Scotland Ave, Albany, NY 12208, USA; dominick.centurioni@health.ny.gov (D.A.C.); stephen.davis@health.ny.gov (S.W.D.); christina.egan@health.ny.gov (C.T.E.); 2 Bacteriology Laboratory, Wadsworth Center, New York State Department of Health, 120 New Scotland Ave, Albany, NY 12208, USA; george.hannett@health.ny.gov (G.E.H.); kimberlee.musser@health.ny.gov (K.A.M.)

**Keywords:** *Clostridium botulinum*, mass spectrometry, toxin, mouse bioassay

## Abstract

Currently, the gold standard method for active botulinum neurotoxin (BoNT) detection is the mouse bioassay (MBA). A Centers for Disease Control and Prevention-developed mass spectrometry (MS)-based assay that detects active BoNT was successfully validated and implemented in a public health laboratory in clinical matrices using the Bruker MALDI-TOF MS (Matrix-assisted laser desorption ionization–time of flight mass spectrometry) Biotyper. For the first time, a direct comparison with the MBA was performed to determine MS-based assay sensitivity using the Bruker MALDI Biotyper. Mice were injected with BoNT/A, /B, /E, and /F at concentrations surrounding the established MS assay limit of detection (LOD) and analyzed simultaneously. For BoNT/B, /E, and /F, MS assay sensitivity was equivalent or better than the MBA at 25, 0.3, and 8.8 mLD_50_, respectively. BoNT/A was detected by the MBA between 1.8 and 18 mLD_50_, somewhat more sensitive than the MS method of 18 mLD_50_. Studies were performed to compare assay performance in clinical specimens. For all tested specimens, the MS method rapidly detected BoNT activity and serotype in agreement with, or in the absence of, results from the MBA. We demonstrate that the MS assay can generate reliable, rapid results while eliminating the need for animal testing.

## 1. Introduction

*Clostridium botulinum* is a Gram-positive, anaerobic, spore-forming, bacillus capable of producing *C. botulinum* neurotoxin (BoNT), a deadly neurotoxin that is the causative agent of the paralytic illness botulism. Currently, seven BoNT serotypes have been identified (BoNT/A–/G) [1]. BoNTs, which can be produced by *C. botulinum*, *C. butyricum*, *C. argentinense*, and *C. baratii*, are translated as 150 kDa heterodimers. This toxic protein is composed of a covalently-linked 100 kDa heavy chain (HC) and a 50 kDa light chain (LC). The HC is responsible for toxin binding and transport, while the LC is responsible for enzymatic activity [2,3]. Upon BoNTs entering neurons, a disulfide bond is reduced, separating the HC and LC and activating the toxin [4]. The zinc-dependent neurotoxin protease inhibits the release of acetylcholine at neuromuscular junctions by cleaving proteins located within the soluble N-ethylmaleimide-sensitive fusion attachment protein receptor (SNARE) complex with high specificity, resulting in flaccid paralysis [5]. Botulism can be caused by ingestion of pre-formed toxin (foodborne), germination of spores within a wound (wound), ingestion of bacterial spores (infant and adult intestinal), or inhalation of spores (inhalational). Infant botulism is the most prevalent form of botulism and is typically associated with type A or B toxin production [6]. Toxin inhalation is extremely rare and would likely be associated with a bioterrorism event. Due to its acute toxicity and potential for misuse, BoNT is classified as a Tier 1 select agent. 

It is essential that neurotoxin detection methods are both sensitive and rapid, as administration of the costly treatment can rely on toxin identification within clinical or food specimens. The current gold standard method for toxin detection is the mouse bioassay (MBA); however, this assay has several limitations. While this method is sensitive, it requires sample volumes of 1 mL per toxin type and, ideally, a total sample volume of over 4 mL. It is not always possible to obtain such large sample volumes, especially when samples originate from infant stool or food remnants. Low available volumes can be especially problematic when dealing with suspected infant botulism cases. This assay can also take considerable time to yield clear results. Once specimens are bound with antitoxins and injected into mice, the mice must be monitored for lethality. Although the MBA may yield positive results within 24 h, it can take four or five days to identify negative or low levels of toxin in specimens. Another shortcoming of the MBA is that other bacteria or microbes present in the specimen can interfere with the assay, resulting in inconclusive results. Interferences from other substances, such as proteases and low-molecular-weight molecules, can be lethal in mice and inhibit the MBA [7,8]. There are also a number of practical issues involved in performing MBA. The upkeep for the mice can be both labor-intensive and costly, as they quickly outgrow their diagnostic size [9]. Also, mice must always be kept on-site in order to begin testing immediately. Finally, this assay requires the euthanization of laboratory animals, which should be avoided when an alternative method is available.

Researchers at the Centers for Disease Control and Prevention (CDC) have developed a rapid specific mass spectrometry (MS)-based method, Endopep-MS, capable of detecting all seven BoNT serotypes and toxin activity when used in conjunction with a high-resolution mass spectrometer. This method, which eliminates many of the drawbacks associated with the MBA, has been previously described and is well-characterized [10,11]. Previously, many clinical and public health laboratories (PHL) were unable to use this method, as it requires the use of a costly high-resolution mass spectrometer, which most did not possess. However, with advancements in MS development and marketing, microbiology laboratories are beginning to acquire these instruments due to their superior ability to identify bacteria and yeast. MS instruments, including the Bruker MALDI-TOF MS (matrix-assisted laser desorption ionization–time of flight mass spectrometry) Biotyper and the bioMérieux Vitek MS, are now being manufactured in size and price ranges that are appealing and obtainable for clinical and PHL that perform microbiological testing. While both of these instruments are excellent at identifying microorganisms from isolates, they have a lower resolution than MS instruments previously used for BoNT identification [12]. These instruments can only be operated in linear positive ion mode, which yields lower resolution spectra when compared to more costly instruments capable of being operated in reflectron mode. However, the Bruker MALDI Biotyper has a number of positive characteristics that outweigh its limitations. It has a smaller footprint, is much less expensive to purchase, and can be easily operated by clinical technicians. It has been used successfully to detect carbapenem resistance in *Bacteroides fragilis* due to the presence of cfiA gene and to detect resistance against β-lactam antibiotics by monitoring the cleave of the β-lactam ring by β-lactamases [13,14,15]. For these reasons, the Bruker MALDI Biotyper was the MS instrument of choice for this study.

This article describes the validation and implementation of the CDC Endopep-MS method using the Bruker MALDI Biotyper and, for the first time, the utilization of the Bruker MALDI Biotyper to identify BoNT in clinical specimens. Overall assay performance and a direct comparison of the MS assay to the MBA will be highlighted for *Clostridium botulinum* neurotoxin types that affect humans (Types A, B, E, and F). 

## 2. Results

### 2.1. Sensitivity and Specificity

In order to determine the sensitivity of the assay, 10-fold serial dilutions of all four toxin types (A, B, E, F) were spiked into HBS-EP buffer, serum, and stool matrices, and analyzed using the Bruker MALDI Biotyper. For each matrix, the limit of detection (LOD) was determined to be the lowest concentration of toxin that produced positive results in all replicates for the MS assay. Samples were considered positive if both daughter peptide substrate fragments corresponding to the correct toxin type were present (Table 1). Sensitivity in buffer, serum, and stool matrices for BoNT/A, /B, /E, and /F were found to be 18, 25, 0.3, and 8.8 mLD_50_, respectively. In situations where only one fragment was above the threshold cutoff, samples were considered indeterminate, and the sample was re-spotted on the MALDI target. Samples that were still indeterminate after re-spotting were processed again if sufficient specimen volume was available. As proteases in the matrices may cause nonspecific cleavage of the substrate, resulting in the observation of a single fragment, presence of both fragments was required for positive identification. In addition, 100% concordance was observed both within and between runs when specimens were spiked near (10X LOD) and above (100X LOD) the LOD which supports the assay being highly reproducible.

The specificity of the assay was determined by testing culture supernatants from enrichment broths of organisms that were genetically related, organisms and toxins that could produce similar symptoms or illness, and organisms that could be present in the clinical specimens received (Table 2). As expected, neurotoxin producing *Clostridium* spp. which included *C. botulinum* and *C. baratii*, were detected by this method. Non-toxigenic strains, other toxins, and culture supernatants from enrichment broths of closely related organisms all produced negative results, indicating the absence of cross-reactivity with the organisms and toxins in the specificity panel.

### 2.2. Method Comparison

To compare this MS-based method to the currently utilized MBA, mice were injected with human serum containing levels of toxin at and around the LOD of this MALDI-TOF MS assay for each toxin type. Mice were monitored for four days, in accordance with the accepted MBA protocol. Using the MS assay, BoNT/E and /F were detected at concentrations 10-fold lower than with the MBA, indicating that the MS assay was more sensitive (Table 3, Table 4, Table 5 and Table 6). No difference in detection was observed between the two test methods for BoNT/B. The LOD of BoNT/A for the MS assay was observed to be 18 mLD_50_, whereas the MBA was detected as low as 1.8 and 18 mLD_50_. This study was limited by the number of mice we could utilize and, thus, the concentrations of toxin were also limited. 

A direct comparison study between methods was also conducted over the course of six months (Table 7). As clinical specimens (*N* = 25) were received for *C. botulinum* neurotoxin testing, they were tested by both the MBA and the MS method. The MS-based method detected BoNT activity and the toxin type that was present within 8 h in all tested specimens that were also positive by the MBA. In multiple cases, MS test results were available within 4 h of receipt of the specimen. Specimens believed to contain high concentrations of BoNT were analyzed after just one hour of incubation, versus the typical 4 h incubation period. It should be noted that if test results were negative after one hour, samples were re-incubated for the remaining 3 h period and tested again. While both methods produced similar results for the specimens tested, only MS-based testing could be conducted for nine out of the 25 samples submitted, since there was not sufficient sample volume to run the MBA. Interestingly, in four of these nine samples, the MS assay was positive for botulinum neurotoxin. 

**Figure 1:**
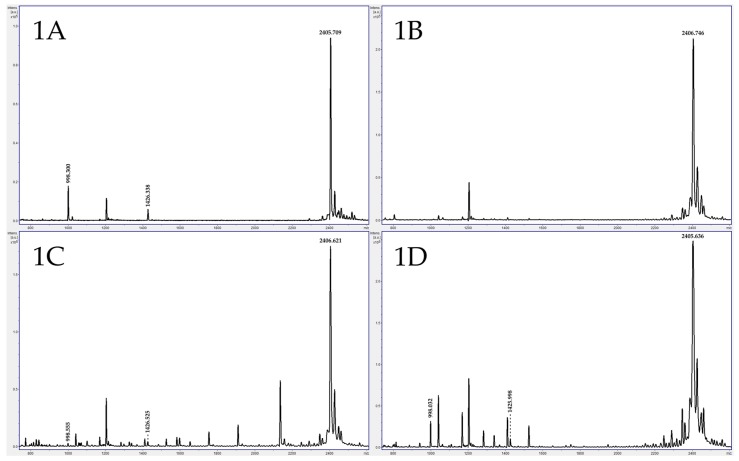
A typical BoNT/A positive control from the Bruker MALDI Biotyper has an intact peptide substrate at 2406 *m*/*z* and two peptide fragments at 998 and 1426 *m*/*z* (**1A**); One patient exhibited negative BoNT/A in serum (**1B**, 14-17944-01); however, a low concentration of BoNT/A was detected by MS in stool from the same patient (**1C**, 14-17944-02), whereas the mouse bioassay was negative for both specimen types. The patient specimen results were later confirmed when the contaminated food the patient ingested was positive for BoNT/A by MS (**1D**, 14-18209) and the mouse bioassay.

All test results were verified by an in-house developed multiplex polymerase chain reaction (PCR) assay capable of detecting BoNT-producing genes within the organism (Table 7 and Table 8). In Table 7, all MS assay results were supported by the real-time assay, except for one patient sample (14-17944-02) in which toxin was present, but PCR was negative. Toxin presence was confirmed by toxin detection obtained by MS, MBA, and real-time PCR for an implicated food product that the sick patient had ingested (Figure 1). Although the PCR assay can detect low levels of organism in clinical specimens, there are cases in which detection of active BoNT is essential. Table 8 includes a comparison of MS assay results to real-time PCR multiplex assay results from testing of specimens (including several animal specimens) received in 2015. All MS results were concordant with the multiplex PCR assay, with the exception of one stool sample (15-45240) that likely had a low toxin level. 

## 3. Discussion

Using the Bruker MALDI-TOF Biotyper, this study implemented the Endopep-MS method and established its ability to identify BoNT in clinical specimens. Our results indicate that this rapid, cost-effective MS-based method can detect BoNT activity with high analytical sensitivity and specificity. With the implementation of this assay, laboratory expenditures for animal care, maintenance, and personnel time can be reduced, while allowing time-sensitive patient results to be available up to four times faster than with the current MBA. 

Our data support the conclusion that the MS assay is at least as sensitive as (if not superior to) the MBA for detecting BoNT/B, /E, and /F. It appears that there may be a slight increase in sensitivity in the MBA for detecting BoNT/A (Table 3). When the method comparison studies were performed, limited toxin concentrations were used to compare methods. The concentration of the spike levels was determined based upon preliminary studies conducted in our laboratory that provided initial LOD information. Based on this information, 10- and 100-fold concentrations above the LOD were chosen to characterize any detection issues arising from a saturation of toxin concentration. We believe that the LOD of the mass spectrometry assay may be much closer than the approximate 10-fold difference that was obtained in our study. We would anticipate that the sensitivity of both assays are actually much less than 10-fold, based on our method comparison studies and the fact that at the 1.8 mLD_50_ there was a positive/negative result in the MBA. To determine the exact difference in detection between the test methods, further studies utilizing additional concentrations need to be completed. However, considering the results from the side-by-side comparison of clinical specimens, it appears that the sensitivity of the MS method for BoNT/A is sufficient to determine the presence of active *C. botulinum* toxin at clinically-relevant levels. 

The MALDI-TOF MS assay is an appealing alternative or adjunctive method to the MBA for several reasons. If patients present with symptoms consistent with botulism, they may be treated without reliance on botulism diagnostic testing. However, patients exhibiting atypical symptomology must rely on botulism testing in order to receive treatment, such as anti-toxin administration. Reduction of testing time to 4–6 h from the 1–4 days typically required by the MBA would allow for more rapid and effective treatment of individuals that have an atypical botulism presentation. Additionally, the MBA is typically performed by the CDC, the Food and Drug Administration, and a few PHL. Only a limited number of PHL are capable of detecting BoNT using the MBA, as the requirements for maintaining an animal care facility are costly and labor-intensive. Additionally, many PHL do not want to maintain a Tier 1 Select Agent registration, which is required for conducting the MBA when the total BoNT concentrations surpass 0.5 mg. The utility of the MALDI-TOF MS assay will provide PHL an alternative method for detecting BoNT, thus greatly expanding the number of laboratories that can perform confirmatory testing. 

MS methods can also successfully identify BoNT activity for the four toxin serotypes (A, B, E, and F) that affect humans, and are particularly useful in cases where the more traditional methods cannot be performed due to potentially disruptive factors, such as the presence of other microorganisms. Additionally, in complex matrices, such as stool, the MS method performs well in situations where the MBA can produce conflicting and/or inconclusive test results. For example, in one case of foodborne botulism, using the MALDI-TOF MS method, we were able to successfully detect active BoNT/A from the primary specimen, although MBA results were indeterminate. The MS results were further confirmed during additional testing of food suspected of being the source of toxin production. MS testing of this suspect food source identified the same toxin type previously confirmed by MS testing from the patient specimen, while the MBA results for both patient and food samples were considered indeterminate. 

The MS-based method also provides a solution to a common problem in testing for botulism. Ideally, patient specimen volume greater than 4 mL total (1 mL per toxin type) is necessary to run the MBA, and without sufficient volume, the MBA cannot be run at all. Botulism, as a paralytic illness, can hinder the collection of primary stool samples. With reduced peristalsis, affected adults and infants may not be able to produce enough of a specimen to be used for testing. However, with the MS-based method, samples can be tested with sample volumes as low as 100 µL per toxin type. Of the specimens submitted to us for botulism testing, nine out of 25 had an unsatisfactory volume for the MBA. The requirement for a large sample volume when testing by the MBA is concerning because, in four of the cases where insufficient specimen volume was submitted, the specimens were positive for BoNT/B by the MS method. These data were confirmed after culturing a *C. botulinum* isolate from the specimen and performing additional MBA and MS analysis. Clearly, assays that can be used on low volume specimens that are commonly received with botulism cases are extremely beneficial, as they can provide essential information for public health personnel and the clinical care of the patient. 

An additional benefit of the MS-based assay is its ability to test serum specimens which cannot be tested by PCR, as the organism is typically not detected in patient serum specimens due to the lack of circulation of *C. botulinum* in the bloodstream. PCR results may also produce confounding results, as some Clostridia produce genes for multiple botulinum neurotoxin serotypes, but will only produce one type of toxin. Also, in some cases where toxin genes are present, toxin may not be produced [16]. There was one such result (14-18209) shown in Table 7 and three samples (15-09115, 15-09115-01, and 16-2332) in Table 8. It has been reported that there are several strains of *C. botulinum* that produce a toxin Type A, yet have a silent B gene present [17]. We have seen this multiple times in the northeast NY in previous years (data not shown). In these cases, the MS assay has proven to be very useful, as it can detect and distinguish which toxin type is being produced by organisms. 

Our data support the use of the MS assay through equivalent detection levels that were obtained for the MS method with respect to the MBA. While the BoNT/A MS sensitivity may be slightly less sensitive, our method comparison study supports testing using the MS assay. Of the three specimens that tested positive for BoNT/A (Table 7), all three were positive utilizing the MS assay; however, only two were positive by the MBA. Table 8 also shows three samples that were positive for BoNT/A by both methods (real-time PCR and MS). These data indicate that the MS assay detects clinically-relevant concentrations of BoNT/A. 

This assay has been shown to be effective in food matrices, making it a useful public health investigative tool. Toxin could potentially be ingested by an individual in a food source where organisms are present in very small numbers or not at all. In this case, a PCR screening assay may not produce positive results, but cannot confirm negative results, leaving it unclear whether the patient is affected by the potent neurotoxin. If sufficient specimen quantities are not available for testing with the current methods, affected individuals may be misdiagnosed and left untreated. In summary, our data provides evidence that the MALDI-TOF MS assay is a sensitive, specific, and accurate alternative to the MBA assay, and that use of the Bruker MALDI Biotyper is an acceptable platform for botulism toxin testing.

## 4. Materials and Methods

### 4.1. Materials and Reagents

Unless indicated otherwise, all chemicals were obtained from Sigma-Aldrich (St. Louis, MO, USA). All peptides were synthesized and purchased from Midwest Biotech Incorporated (Fishers, IN, USA). Peptide sequences can be found in Table 1 [18]. Isolates and toxins used to establish assay specificity were obtained from the Wadsworth Center Culture Collection, American Type Culture Collection (ATCC), Vector Laboratories, or Toxin Technologies. BoNT/A, /B, /E, and /F complexes were purchased as 1 mg/mL stocks with toxin concentrations as follows: BoNT/A at 3.60 × 10^7^ mLD_50_/mL, BoNT/B at 1.25 × 10^7^ mLD_50_/mL, BoNT/E at 3.00 × 10^7^ mLD_50_/mL, and BoNT/F at 4.40 × 10^6^ mLD_50_/mL from Metabiologics (Madison, WI, USA). As toxin concentrations were above the exempt maximum concentration of 0.5 mg, a Tier 1 Select Agent laboratory was necessary to perform all experiments. Previously described antibodies were provided by James D. Marks, MD, Ph.D. (University of California, San Francisco, CA, USA) and Suzanne Kalb, PhD (CDC, Atlanta, GA, USA) [19,20,21]. MS peptide calibrant was obtained from Bruker Daltonics (Billerica, MA, USA). Magnetic Dynabeads were obtained from ThermoFisher Scientific (Grand Island, NY, USA). Donated stool had all identifying information removed. De-identified human serum and stool for spiking studies were obtained for these studies with donor consent following a New York State Department of Health Institutional Review Board-approved protocol (03-037). Serum for protease inhibition was obtained from Equitech-Bio (Kerrville, TX, USA). Wadsworth Center Institutional Animal Care and Use Committee approval was obtained for all studies involving mice (10-112, 2010; 13-112, 2013).

### 4.2. Sample Preparation

Stool specimens were prepared by transferring 1–2 g of specimen into a pre-weighed stomacher bag. Gelatin diluent buffer was added to the bag at a 1:1 ratio (stool:buffer). Using a micro-stomacher, specimens were processed at 200 rpm for 5 min. The contents of the bag were placed into a conical tube and centrifuged at 12,000× *g*, 4 °C for 10 min. The supernatant was retained for processing.

### 4.3. Mass Spectrometry Assay

IgG beads were coupled and cross-linked with sulfo-NHS-Biotin (0.522 mM) and the appropriate toxin serotype antibody (2 µg). As a singleplex, each sample contained 200 µL HBS-EP buffer, 200 µL serum, 50 µL 10x PBST, and 20 µL of the appropriate toxin type IgG beads. Patient specimen (100 µL) or negative control matrix was added to each sample. Spike patient specimens used for the sensitivity studies were prepared by serially diluting 2 µL of the concentrated toxin stocks described above in 18 µL of MS-grade water. These stocks were spiked (2 µL) into each matrix (98 µL) to obtain the desired final concentration. Positive control was prepared by diluting toxins with LC-MS-grade water in Lo-bind protein tubes to a final concentration of 10^3^–10^4^ mLD_50_/mL. Two microliters of the prepared toxin stocks were added into negative control matrix (100 µL) to be used as a positive control. Microcentrifuge tubes containing the above samples were briefly vortexed before being placed on a shaker set to 1450 rpm for 1 h. After shaking, samples were placed onto a magnetic capture apparatus. Once the beads had settled, samples were washed with 1 mL aliquots four times, twice with 2 M NaCl/PBST, then twice with HBS-EP. Finally, all remaining solution was removed from the beads and 50 µL of LC/MS-grade water was added to each sample.

Samples were transferred to a 96-well thermocycler plate on top of a 96-well polymerase chain reaction (PCR) plate magnet from Thermofisher Scientific (Grand Island, NY, USA). After removing the water from the magnetically-separated beads, 18 µL of reaction buffer (10 mM dithiothreitol, 200 mM HEPES, 10 µM zinc chloride, and 1 mg/mL bovine serum albumin) and 2 µL of the corresponding peptide substrate (0.1 mM) were added to the appropriate samples (Table 1). Samples were incubated at 37 °C for 4 h. 

### 4.4. MALDI-TOF MS Detection

After the incubation period, the thermocycler plate was placed on a magnetic plate magnet. A matrix solution was prepared in a separate tube by adding 2 µL of supernatant contained in the thermocycler plate to 18 µL of alpha-cyano-4-hydroxycinnamic acid (CHCA) matrix (5 mg/mL CHCA in 50% acetonitrile, 0.1% TFA, 1 mM ammonium citrate). After vortexing the mixture, 0.7 µL was spotted onto a steel target plate and allowed to air dry completely. Mass spectra of each spot were obtained by scanning from 500 to 5500 *m*/*z* in MS-positive ion linear mode on a Bruker MALDI Biotyper. The instrument uses a nitrogen laser at 337 nm. Acceleration voltage was set to 20 kV and each spectrum was an average of 1000 laser shots. To be considered positive, both daughter fragment peaks were required to have a signal-to-noise (S/N) ratio above 2.0 for type A, B, and F. Type E had two daughter fragments at a mass-to-charge (*m*/*z*) of 1132 and 2500 and was required to have an S/N ratio greater than 6.0 and 10.0, respectively. In addition, the relative intensity of all target peaks was required to be above 500 absorbance units (AU) for type A, B, E, and F.

### 4.5. Mouse Bioassay

The MBA was performed according to the procedure of Dowell [22]. Briefly, an equal volume of gelatin diluent (*w*/*v*) was added to stool. The stool was thoroughly mixed, and refrigerated overnight at 5 °C. The stool extract was centrifuged (12,350× *g*, 5 °C, 20 min) and the supernatant tested for BoNT. The supernatant was split into two portions and an aliquot was neutralized with type-specific antitoxin (Antitoxin was provided by the CDC, Atlanta, GA, Georgia). Intraperitoneal injection of Swiss Webster mice was performed using either an un-neutralized or neutralized extract. Mice with an approximate mass of 20 g were injected with 0.4 mL of human serum spiked with purified BoNT complex and observed for at least four days. Two mice per toxin type (A/B/E/F) were injected with toxin, while two control mice were injected with 0.5 mL of negative human serum. Specimens were considered positive for the presence of BoNT if the mice receiving the neutralized extract survived and mice receiving the un-neutralized extract either developed signs of botulism intoxication or expired. Mice were euthanized if signs of botulism developed. To reduce the number of mice required for spiking study comparisons, a modified version of this protocol did not include the use of neutralized extracts. 

### 4.6. Extraction and Multiplex PCR

Clinical stool specimens were obtained from the New York State Department of Health Bacteriology Laboratory (Albany, NY, USA). Pretreatment to remove solid materials from stool specimens was required before performing the extraction as per the manufacturer. Stool specimens and environmental samples were diluted in Tris EDTA buffer to obtain 200 µL of liquid content, mixed thoroughly and heat-treated at 95 °C for 30 min. The specimen was then centrifuged for 10 s at 12,000× *g* and the supernatant was recovered. The supernatant was utilized for nucleic acid extraction by either manual or automated methods. Manual extraction was performed using the Epicentre MasterPure complete DNA and RNA Purification Kit (Madison, WI, USA) with the modification of a 30 min lysis step followed by filtering in a Bio 101 spin module column (Palo Alto, CA, USA) to remove unlysed spores. 

Automated DNA extraction of specimens was performed utilizing the Roche MagNA Pure LC and Total Nucleic Acid Extraction Kit I (Indianapolis, IN, USA). Kit I contains wash buffers for removing PCR inhibitors, salts and proteins, lyses and binding solution, proteinase K, magnetic beads for binding of DNA, and an elution buffer. Post-extraction filtering in a spin module column was also performed as stated above. 

Both of the multiplex reactions consisted of 25 μL reaction volumes and utilized the Roche LightCycler^®^ FastStart DNA Master Hybridization Probes kit (Indianapolis, IN, USA). Each reaction mixture had the final concentration of the following reagents: 1 × LightCycler^®^ FastStart DNA Master Hybridization Probes mix, 4 mM MgCl_2_, 450 nM forward and reverse primers, 125 nM probes, and 5 μL of sample volume. 

All real-time PCR reactions were performed using the Applied Biosystems Inc. (Carlsbad, CA, USA) 7000 or 7500 Fast Sequence Detection System with SDS software version 1.4.0 (Waltham, MA, USA). The real-time PCR reactions were performed using standard cycling conditions that included a 95 °C denaturation for 10 min, followed by 45 cycles of 95 °C for 15 s and 60 °C for 60 s, with no passive reference dye utilized. Each run was analyzed separately for each probe dye utilized. 

### 4.7. Assay Validation

This endopeptidase-based method was validated by determining the analytical specificity, analytical and clinical sensitivity, inter- and intra-assay reproducibility, and accuracy in clinical matrices. Serum and stool matrices were validated for the identification of BoNT types A, B, E, and F. Since sufficient specimen quantities are not always available, spiked samples were used when validating this assay. De-identified negative human serum and stool were collected and spiked with purified BoNT complex prepared to the appropriate concentrations in LC-MS grade water. A method comparison to the MBA was performed. Mice were injected with spiked serum at concentrations at and around the limit of detection (LOD) of our MS-based method. In addition, clinical specimens received during a six-month time period were tested side-by-side with an in-house developed multiplex PCR assay and the MBA method. 

## Figures and Tables

**Table 1:** Peptide substrate amino acid sequences with respective mass cleavage products.

Toxin Type	Peptide Sequence	Intact Substrate Mass/Charge (Da)	Cleavage Fragment 1 (Da)	Cleavage Fragment 2 (Da)
BoNT A	Ac-RGSNKPKIDAGNQRATRXLGGR-NH_2_^1^	2406	998	1426
BoNT B	LSELDDRADALQAGASQFESSAAKLKRKYWWKNLK	4026.8	1759	2283
BoNT E	H_2_N-WWWAKLGQEIDTRNRQKD(hR)IMAKADSNKR- NH_2_	3615	1132	2500
BoNT F	TSNRRLQQTQAQVDEVVDIMRVNVDKVLERDQKLSELDDRADAL	5112	1345	3783

^1^ X is Norleucine.

**Table 2:** Mass spectrometry specificity studies.

Organism/Toxin	Source	Peptide Substrate Type A	Peptide Substrate Type B	Peptide Substrate Type E	Peptide Substrate Type F
*C. botulinum* (A)	A219-A76	+	−	−	−
*C .botulinum* (B)	12-000-19474	−	+	−	−
*C .botulinum* (E)	1987-818	−	−	+	−
*C. botulinum* (F)	ATCC 35415	−	−	−	+
*C. botulinum* (A and B) ^1^	WC	+	+	−	−
*C. botulinum* (E) (Non-toxigenic)	BAC-08-38772	−	−	−	−
*C. baratii* (F)	BAC-07-4010	−	−	−	+
*C. baratii* (Non-toxigenic)	ATCC 7659	−	−	−	−
*C. difficile*	2014-30621	−	−	−	−
*C. sporogenes*	CDC 1967	−	−	−	−
*C. novyi*	CDC 1976	−	−	−	−
*C. subterminale*	2012-39165	−	−	−	−
*C. perfringens*	ATCC 13124	−	−	−	−
*C. butyricum*	1972-B1612	−	−	−	−
*C. tetani*	CDC 14339	−	−	−	−
Shiga Toxin Producing *E.coli* (O157:H7)	ATCC 700728	−	−	−	−
*V.parahaemolyticus*	ATCC 49529	−	−	−	−
Ricin A Chain	Vector Labs	−	−	−	−
*S. aureus* Enterotoxin B	Toxin Technologies	−	−	−	−
*C. jejuni*	ATCC 33560	−	−	−	−

^1^ Wadsworth Center (WC) specimen was spiked with two toxins, BoNT/A and /B.

**Table 3:** Side-by-side comparison of the mouse bioassay and the mass spectrometry method for BoNT/A in human serum.

BoNT Type A (mLD_50_)	MALDI-TOF MS		Mouse Bioassay	
	Replicate 1	Replicate 2	Mouse 1	Mouse 2
72	Positive	Positive	Expired	Expired
36	Positive	Positive	Expired	Expired
18	Positive	Positive	Expired	Expired
1.8	Negative	Negative	Expired	Survived
0.18	Negative	Negative	Survived	Survived
0.018	Negative	Negative	Survived	Survived
Negative	Negative	Negative	Survived	Survived

**Table 4:** Side-by-side comparison of the mouse bioassay and the mass spectrometry method for BoNT/B in human serum.

BoNT Type B (mLD_50_)	MALDI-TOF MS		Mouse Bioassay	
	Replicate 1	Replicate 2	Mouse 1	Mouse 2
100	Positive	Positive	Expired	Expired
50	Positive	Positive	Expired	Expired
25	Positive	Positive	Expired	Expired
2.5	Negative	Negative	Survived	Survived
0.25	Negative	Negative	Survived	Survived
0.025	Negative	Negative	Survived	Survived
Negative	Negative	Negative	Survived	Survived

**Table 5:** Side-by-side comparison of the mouse bioassay and the mass spectrometry method for BoNT/E in human serum.

BoNT Type E (mLD_50_)	MALDI-TOF MS		Mouse Bioassay	
	Replicate 1	Replicate 2	Mouse 1	Mouse 2
15.0	Positive	Positive	Expired	Expired
7.5	Positive	Positive	Expired	Expired
3.0	Positive	Positive	Sacrificed ^1^	Survived
0.3	Positive	Positive	Survived	Survived
0.03	Negative	Negative	Survived	Survived
0.003	Negative	Negative	Survived	Survived
Negative	Negative	Negative	Survived	Survived

^1^ One mouse with a dose of 3 mLD_50_ showed signs of botulism intoxication (wasp-waist, labored breathing, impaired locomotion, ruffled fur) and was sacrificed 22 h post inoculation.

**Table 6:** Side-by-side comparison of the mouse bioassay and the mass spectrometry method for BoNT/F in human serum.

BoNT Type F (mLD_50_)	MALDI-TOF MS		Mouse Bioassay	
	Replicate 1	Replicate 2	Mouse 1	Mouse 2
352	Positive	Positive	Expired	Expired
176	Positive	Positive	Expired	Expired
88	Positive	Positive	Expired	Survived
8.8	Positive	Positive	Survived	Survived
0.88	Negative	Negative	Survived	Survived
0.088	Negative	Negative	Survived	Survived
Negative	Negative	Negative	Survived	Survived

**Table 7:** Mouse bioassay, MALDI-TOF MS assay, and multiplex PCR assay method comparison utilizing clinical specimens.

Specimen ID Number	Specimen Type	PCR Results ^1^	MS Results (ABEF)	Mouse Bioassay Results ^1^
14-17523	Stool	Negative	Negative	N/A
14-17280-01	Stool	Negative	Negative	N/A
14-17280-02	Serum	N/A	Negative	N/A
14-16815	Enema (Stool)	Positive, B	Positive, B	Positive, B
14-15986-01	Stool	Negative	Negative	Negative
14-15986-02	Serum	N/A	Negative	Negative
14-40315	Stool	Positive, B	Positive, B	N/A
14-40316	Stool	Positive, B	Positive, B	N/A
14-22234	Enema (Stool)	Positive, B	Positive, B	N/A
14-19626-01	Enema (Stool)	Negative	Negative	N/A
14-20126-01	Stool	Positive, B	Positive, B	N/A
14-17934-01	Serum	N/A	Negative	Negative
14-17934-02	Stool	Negative	Negative	Negative
14-17944-01	Serum	N/A	Negative	Negative
14-17944-02	Stool	Negative	Positive, A	Negative
14-18209	Spaghetti sauce with peas and meat	Positive, A & B	Positive, A	Positive, A
14-18198	Spaghetti sauce	Negative	Negative	N/A
A219-A76	Broth filtrate	Positive, A	Positive, A	Positive, A
12-19474	Broth filtrate	Positive, B	Positive, B	Positive, B
12-17942	Broth filtrate	Positive, B	Positive, B	Positive, B
1987-818	Broth filtrate	Positive, E	Positive, E	Positive, E
2008-38772	Broth filtrate	Negative	Negative	Negative
ATCC 35415	Broth filtrate	Positive, F	Positive, F	Positive, F
2007-4010	Broth filtrate	Positive, F	Positive, F	Positive, F
ATCC 7659	Broth filtrate	Negative	Negative	Negative

^1^ Specimens that were not tested are listed as N/A. Sera were not tested by PCR because they were not expected to contain *C. botulinum*. Specimens that contained insufficient specimen volume were not tested by the mouse bioassay.

**Table 8:** Method comparison of MALDI-TOF MS assay detecting toxin and multiplex PCR assay detecting toxin producing genes of *Clostridium* spp. utilizing human and animal specimens.

Specimen ID Number	Specimen Type	PCR Results	MS Results (ABEF)
15-09115	Stool	Positive, A + B	Positive, A
15-10496	Probiotics, Broth filtrate	Negative	Negative
15-09115-01	Broth filtrate	Positive, A + B	Positive, A
14-40280-02	Intestine Contents	Positive, E	Positive, E
15-09124	Stomach Contents	Negative	Negative
15-11314	Loop of Bowel	Negative	Negative
15-11315	Intestine Contents	Negative	Negative
15-45240	Enema (Stool)	Positive, B	Negative
15-67526	Stool (Enrichment)	Negative	Negative
15-68267	Stool	Negative	Negative
15-58-01	Isolate	Negative	Negative
15-58-02	Isolate	Negative	Negative
15-112	Stomach Contents	Positive, E	Positive, E
15-113	Intestine Contents	Positive, E	Negative
15-115	Liver	Negative	Positive, E
15-117	Intestine Contents	Positive, E	Positive, E
15-129	Stomach Fluid	Positive, E	Positive, E
15-130	Intestine Contents	Positive, E	Positive, E
15-131	Stomach Contents	Negative	Positive, E
16-2332	Stool	Positive, A + B	Positive, A
16-2476	Stool	Positive, B	Positive, B
15-72552	Serum	Negative	Negative
15-72549	Gastric Contents	Negative	Negative
16-5619-01	Stool	Positive, B	Positive, B
16-5619-02	Stool	Positive, B	Positive, B
